# Efficacy and safety of fluocinolone acetonide intravitreal implant (0.2 µg/day) in patients with post-surgical inflammation associated with macular edema: a case series study

**DOI:** 10.1186/s12348-025-00503-8

**Published:** 2025-08-16

**Authors:** Maria Madeira, Ana Cabugueira, Helena Urbano, Miguel Cordeiro, Marta Guedes

**Affiliations:** Ophthalmology Department, West Lisbon Local Health Unit (Unidade de Saúde Local de Lisboa Ocidental), Rua da Junqueira, 126, Lisbon, 1349-019 Portugal

**Keywords:** Postoperative cystoid macular edema, Inflammation, Fluocinolone acetonide intravitreal implant, ILUVIEN, Intraocular inflammation, Intraocular pressure

## Abstract

**Background:**

Postoperative cystoid macular edema (PCME) is a primary cause of reduced vision following both cataract and/or vitreoretinal surgery, which may spontaneously resolve. This study aimed to evaluate the effectiveness and safety of the fluocinolone acetonide intravitreal (FAc) implant (0.2 µg/day) in patients with refractory PCME.

**Methods:**

Retrospective, non-interventional, and single center study conducted on patients with PCME treated with 0.2 µg/day FAc implant. All the patients received previous treatment with topical corticosteroids, nonsteroidal anti-inflammatory drugs (NSAIDs), triancinolone injection and dexamethasone implant. The primary end-points were the mean change in best-corrected-visual-acuity (BCVA) and the proportion of patients gaining ≥ 15 letters from baseline to the last follow-up visit. The secondary endpoints included the mean CRT reduction and the mean intraocular pressure (IOP) during the 36 months study period.

**Results:**

Eight eyes from 8 patients were included in the study. Median (95% Confidence-interval) BCVA was significantly improved from 60.0 (50.05–69.95) letters at baseline to 80.15 (77.25-85.00) letters at month-36, *p* = 0.043. At the last follow-up visit, 5 (62.5%) eyes gained ≥ 15 letters, without any eye experiencing a loss of BCVA compared to baseline. There was significant CRT reduction from baseline (median: 497.5 μm; 95%CI: 380.0–596.0 μm) to month-36 (Median: 252.0 μm; 95%CI: 242.0–268.0 μm); *p* = 0.012. Regarding safety, IOP remained stable from baseline (median: 14.5mmHg; 95%Confidence-interval: 12.0–23.0 mmHg) to the last follow-up visit (median: 13.5mmHg; 95% Confidence-interval: 9.0–19.0 mm Hg); *p* = 0.123.

**Conclusions:**

The FAc implant significantly improved both visual and anatomic outcomes, and was effective in preventing recurrences, while maintaining a reasonable safety profile, in PCME refractory to intravitreal triancinolone and dexamethasone.

## Introduction

Postsurgical cystoid macular edema (PCME) is a well-documented macular complication that can occur after different ocular procedures, such as cataract and vitreoretinal surgeries, which can negatively impact visual outcomes [1–3]. Although some cases resolve spontaneously, persistent PCME detrimentally impacts the quality of patients’ vision, posing a therapeutic challenge for ophthalmologists and carrying substantial financial implications for the healthcare system [4, 5].

The overall reported incidence from historical papers of angiographic PCME is approximately 20–30%, while the incidence of clinical PCME in more contemporary studies with phacoemulsification ranges from 0.2 to 2.35% [6, 7]. Regarding vitreoretinal procedures, the incidence of PCME has been reported to be approximately 5% for vitreous floaters; 10–25% for rhegmatogenous retinal detachment, and 13–47% for epiretinal membrane [7, 8].

Although PCME was described many years ago, its underlying pathophysiology remains uncertain, with numerous proposed mechanisms. Factors contributing to its development include inflammation, vascular instability, vitreomacular tractions, and light toxicity [1–3]. Amongst them, inflammation can be considered as a pivotal factor in the onset of PCME as different molecules and inflammatory mediators are released in response to surgical trauma. These mediators disrupt the blood-retinal barrier, leading to increased permeability of capillaries, ultimately culminating in the accumulation of intraretinal and/or subfoveal fluid around the perifoveal area [2, 3, 9, 10].

Various local (diabetes, previous diagnosis of epiretinal membrane, uveitis, retinal vein occlusion); systemic (diabetes mellitus and systemic hypertension), and surgery-related conditions (capsule rapture, vitreous loss, additional trauma during the surgery) have shown to increase the risk of PCME [2, 3, 8–10].

Treatment options for PCME lack standardization and managing chronic and recalcitrant PCME remains a clinical challenge [2, 3]. First-line treatment typically involves the use of topical corticosteroids and nonsteroidal anti-inflammatory drugs (NSAIDs). If these prove ineffective, secondary treatment options may include intravitreal injections of vascular endothelial growth factor inhibitors (anti-VEGF) or sustained-released intravitreal corticosteroids implants [2, 3].

The intravitreal fluocinolone acetonide (FAc) implant is currently indicated for treating persistent diabetic macular edema and for preventing the recurrence of non-infectious uveitis affecting the posterior segment [11]. In such conditions, its therapeutic effects can last up to 3 years [12, 13].

The evidence assessing the effect of FAc implant in patients with PCME is limited [13–24], with different studies reporting samples of 1 to 49 eyes. Considering this, the main objective of this case series was to evaluate the effectiveness and safety of the FAc implant (0.2 µg/day) in managing post-surgical inflammation associated with PCME.

## Methods

### Study design

Retrospective, non-interventional, and single center case series conducted at the Ophthalmology Department of West Lisbon Local Health Unit, in Lisbon, Portugal. The medical records of patients treated in our center were reviewed. Inclusion criteria were: Eyes diagnosed with a history of post-surgical inflammation (> 6 months) PCME after pars-plana vitrectomy (PPV) and/or cataract surgery; who were either refractory or had an insufficient/short response to previous topical corticosteroids, nonsteroidal anti-inflammatory drugs (NSAIDs), triancinolone injection and dexamethasone implant. This case series adheres to the guidelines for human studies and was conducted ethically in accordance with the principles outlined in the Declaration of Helsinkiand all applicable country-specific regulations governing the conduct of clinical research, depending on which provided greater protection to the individual. Any potentially identifying information has been encrypted or omitted, as necessary, to ensure the anonymity of individuals involved in the study.

### Study participants

Patients with refractory and chronic PCME following an ophthalmic procedure, either cataract or vitreoretinal surgery, who received treatment with a 0.2 µg/day FAc implant were included.

### Study variables

All the demographic and clinical data have been obtained from the medical charts. Anterior chamber (AC) cells, AC flare, vitreous haze, as well as the presence of intraretinal cysts, hyperreflective foci (HRF), and subretinal fluid (SRF) were documented when PCME was diagnosed and at the time of FAc implant injection (baseline). Measurements of best corrected visual acuity (BCVA), central retinal thickness (CRT), macular volume (MV), and intraocular pressure (IOP) were conducted at the time of FAc implant injection (baseline) and at month 1, month 3, and, subsequently, every 3 months until month 36.

### Outcomes

The primary endpoints were the median change in BCVA and the proportion of patients gaining ≥ 15 letters from baseline to the month-36 visit. The secondary endpoints were the median CRT reduction; the proportion of eyes achieving a CRT ≤ 280 μm; the proportion of eyes achieving a CRT reduction ≥ 25% from baseline; mean intraocular pressure (IOP); proportion of eyes with IOP increase ≥ 5 mmHg, ≥ 10 mmHg, or ≥ 20 mmHg; need of IOP lowering surgery; and the need of adjuvant therapy for PCME.

### Statistical analysis

Statistical analysis was performed using SPSS Inc software version 29.0.1.0 and Excel version 16.86. The last-observation-carried-forward method was used to impute missing data. Descriptive statistics median [95% confidence interval (CI) and range] was used to report demographic and ocular characteristics.

The Wilcoxon test was used to assess the differences in BCVA, CRT, and IOP between baseline and the different follow-up visits. Statistical significance was taken as p-value < 0.05.

## Results

Eight eyes from 8 patients were included in the study. The median (95%CI) age was 80.0 (77.0 to 94.0) years (range: 57.0 to 94.0 years), and 4 (50.0%) patients were women. The main baseline demographic and clinical characteristics are shown in the Table 1.

**Table 1:** Main baseline demographic and clinical characteristics of the study sample

	*N* = 8 eyes
Age, years	
Median (95%CI)	80.0 (77.0 to 94.0)
Range	57.0 to 94.0
Sex, n (%)	
Women	4 (50.0)
Men	4 (50.0)
Lens status, n (%)	
Pseudophakic	8 (100.0)
BCVA, ETDRS letters	
Median (95%CI)	60.0 (55.05 to 69.95)
Range	50.05 to 80.15
CRT, µm	
Median (95%CI)	497.5 (380.0 to 596.0)
Range	361.0 to 660.0
Macular volume, mm^3^	
Median (95%CI)	9.19 (8.70 to 10.76)
Range	8.48 to 10.76
Glaucoma, n (%)	
No	7 (87.5)
Yes	1 (12.5)
IOP lowering medication, n (%)	
No	1 (12.5)
Yes	7 (87.5)
IOP, mmHg	
Median (95%CI)	14.5 (12.0 to 23.0)
Range	12.0 to 26.0

*CI* Confidence interval, *PSME* Postsurgical macular edema, *BCVA* Best corrected visual acuity, *ETDRS* Early Treatment Diabetic Retinopathy Study, *CRT* Central retinal thickness, *IOP* Intraocular pressure

All the eyes enrolled in the study underwent cataract surgery, with 2 requiring anterior vitrectomy intraoperatively and 1 necessitating posterior vitrectomy for a luxated lens fragment in a subsequent surgery. The median time from diagnosis to FAc implant was 28.0 (95%CI: 19.0 to 36.0) months. At baseline, two eyes had AC cells and/or flare, respectively. All the eyes presented intraretinal cysts, whilst 4 eyes presented HRF and/or SRF. At baseline, all the eyes have been previously treated with topical NSAIDs, intravitreal injections of triamcinolone, and dexamethasone intravitreal implant. Table 2 shows the main baseline PCME characteristics.

**Table 2:** Main ophthalmic characteristics of the study sample

Study variable	Sample: 8 eyes	
	At diagnosis	At baseline
Length of PSME, months		
Median (95%CI)	3.5 (3.0 to 7.0)	28.0 (19.0 to 36.0)
Range	3.0 to 12.0	11.0 to 57.0
AC Cells, n (%)		
No	3 (37.5)	6 (75.0)
Yes	5 (62.5)	2 (25.0)
AC Flare, n (%)		
No	3 (37.5)	6 (75.0)
Yes	5 (62.5)	2 (75.0)
Vitreous haze, n (%)		
No	5 (62.5)	7 (87.5)
Yes	3 837.5)	1 (12.5)
HRF, n (%)		
No	5 (62.5)	4 (50.0)
Yes	3 (37.5)	4 (50.0)
Cysts, n (%)		
No	0 (0.0)	0 (0.0)
Yes	8 (100.0)	1 (100.0)
SRF, n (%)		
No	2 (25.0)	4 (50.0)
Yes	6 (75.0)	4 (50.0)
Previous treatments, n (%)^a^		
Topical NSAIDs	8 (100.0)	
Topical steroids	7 (87.5)	
Systemic steroids	4 (50.0)	
Oral acetazolamide	1 (12.5)	
Intravitreal triamcinolone	8 (100.0)	
Intravitreal DEX-i	8 (100.0)	
Intravitreal anti-VEGF	4 (50.0)	
Focal laser^b^	1 (12.5)	
Cataract surgery, n(%)		
No	0 (0.0)	
Yes	8 (100)	
Anterior vitrectomy, n (%)		
No	6 (75.0)	
Yes	2 (25.0)	
Posterior vitrectomy n (%)		
No	7 (87.5)	
Yes	1 (12.5)	

*CI* Confidence interval, *PSME* Postsurgical macular edema, *AC* Anterior chamber, *HRF* Hyperreflective Foci, *SRF* Subretinal fluid, *NSAIDs* Nonsteroidal anti-inflammatory drugs, *DEX-i* Dexamethasone intravitreal implant, *anti-VEGF* Vascular endothelial growth factor inhibitor

^a^An eye may have received more than one type of treatment

^b^Inferior-temporal perimacular focal laser in the right eye

### Best corrected visual acuity

**Fig. 1:**
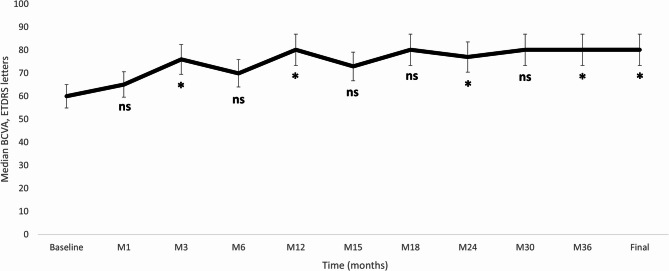
Overview of the best corrected visual acuity (BCVA) throughout the study follow-up. The vertical bars represent the 95% confidence interval. *P* values were calculated with the Wilcoxon test. BCVA: Best corrected visual acuity; ETDRS: Early Treatment Diabetic Retinopathy Study; M: Month; ns: Not significant. *p < 0.05

Median BCVA was significantly improved from 60.0 (50.05 to 69.95) letters at baseline to 80.15 (77.25 to 85.00) letters at month-36, *p* = 0.043. Figure 1 shows the median BCVA at baseline and at the different time-points measured. At the last follow-up visit, the proportion of eyes with a BCVA improvement ≥ 5, ≥ 10, and ≥ 15 ETDRS letters were 75% (6/8), 62.5% (5/8), and 62.5% (5/8), respectively; without any eye experiencing a loss of BCVA compared to baseline values.

### Anatomic outcomes

**Fig. 2:**
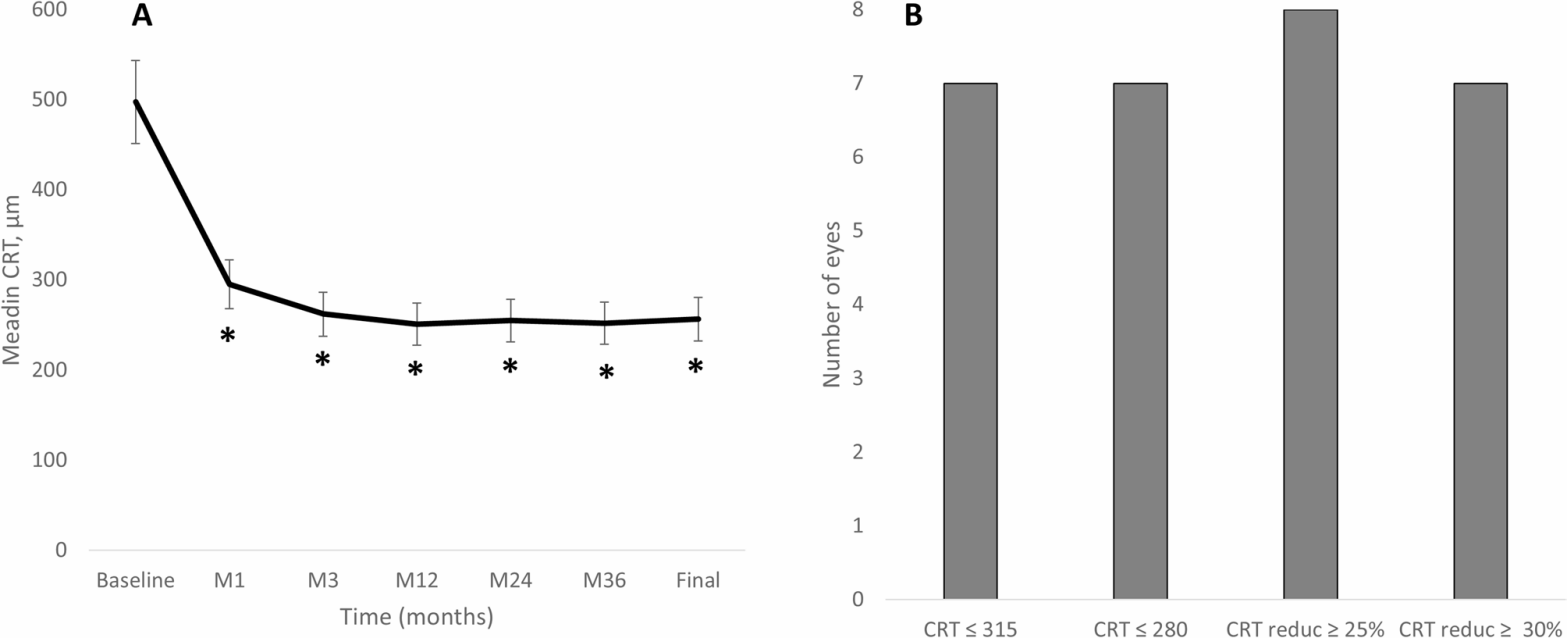
Overview of the central retinal thickness outcomes. **A** Median central retinal thickness throughout the study follow-up. The vertical bars represent the 95% confidence interval. P values were calculated with the Wilcoxon test. **B** Number of eyes reaching a given CRT target at the last follow-up visit. CRT: Central retinal thickness; M: Month; ns: Not significant, reduc: Reduction. *p < 0.05

**Fig. 3:**
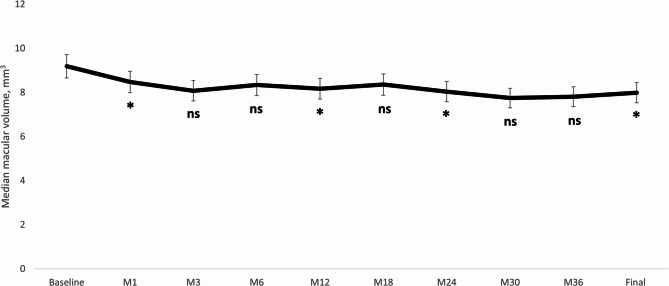
Overview of the macular volume throughout the study follow-up. The vertical bars represent the 95% confidence interval. P values were calculated with the Wilcoxon test. BCVA: Best corrected visual acuity; ETDRS: Early Treatment Diabetic Retinopathy Study; M: Month; ns: Not significant. *p < 0.05

The median changes in CRT and MV throughout the study follow-up are shown in Fig. 2. There was a significant CRT reduction from baseline (median: 497.5 μm; 95%CI: 380.0 μm to 596.0 μm) to month-36 (Median: 252.0 μm; 95%CI: 242.0 μm to 268.0 μm); *p* = 0.012 (Fig. 2A). At the last follow-up visit, 7 (87.5%) eyes presented a CRT ≤ 280 μm. Regarding CRT reduction, 8 (100.0%) eyes achieved a CRT reduction ≥ 25% and 7 (87.5%) eyes a CRT reduction ≥ 30% (Fig. 2B). Similarly, MV was significantly reduced from 9.19 (8.70 to 10.76) mm^3^ at baseline to 7.99 (7.54 to 8.48) mm^3^ at the last follow-up visit (*p* = 0.018) (Fig. 3).

### Safety

**Fig. 4:**
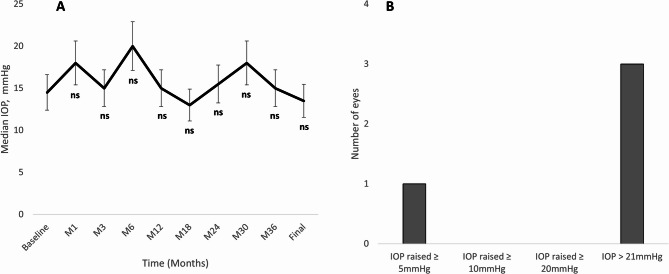
Overview of the intraocular pressure-related outcomes. **A** Median intraocular pressure throughout the study follow-up. The vertical bars represent the 95% confidence interval. *P* values were calculated with the Wilcoxon test. **B** Number of eyes reaching a given intraocular pressure target. IOP: Intraocular pressure; M: Month; ns: Not significant

There were no significant changes in IOP from baseline (median: 14.5 mmHg; 95%CI: 12.0 mmHg to 23.0 mmHg) to the last follow-up visit (median: 13.5 mmHg; 95%CI: 9.0 mmHg to 19.0 mm Hg); *p* = 0.123. As shown in Fig. 4A, there were no changes in IOP throughout the study follow-up. Over the course of the study, one eye experienced an IOP increase of ≥ 5 mmHg, with no eyes experiencing an IOP increase of ≥ 10 mmHg or ≥ 20 mmHg (Fig. 4B). Three eyes experienced an IOP > 21 mmHg at some point during the study follow-up.

At baseline, the median number of ocular hypotensive medications was 2.0 (2.0 to 3.0) drugs; while at the last follow-up visit the number of IOP lowering medications was 1.0 (1.0 to 2.0) drugs. One eye with a prior glaucoma diagnosis underwent trabeculectomy post- FAc implant. No other adverse events related to the FAc implant were observed.

## Discussion

This study retrospectively assessed the efficacy and safety outcomes of FAc implant in patients with refractory PCME under real-world conditions. According to our study results, FAc implant led to a significant improvement in BCVA and reduction in CRT in this patient cohort. Furthermore, 5 out of 8 eyes (62.5%) demonstrated a BCVA improvement ≥ 15 ETDRS letters and, 7 out of 8 eyes (87.5%) experienced a CRT reduction ≥ 30%.

To date, consensus regarding the treatment of PCME remains elusive. Managing post-surgical inflammation, it is often intricate due to its recurrent nature. The current available treatment options comprise topical steroid NSAID drops; subconjunctival or subtenon injections of triamcinolone acetonide; intravitreal injections of anti-VEGF; and intravitreal injections of sustained released intravitreal implants [2, 3].

The use of sustained-release intravitreal implants provides the benefit of extended drug delivery, ensuring longer-term efficacy. Dexamethasone intravitreal implant has shown good efficacy in patients with PCME. However, such effect was reported in small case series and case reports [25–27]. In addition, the EPISODIC-2 study reported that 63% of patients require multiple injections of the dexamethasone implant to effectively manage PCME [28]. Accordingly, in our study, all eyes had previously received multiple treatments, including topical NSAIDs, intravitreal triamcinolone acetonide injections, and dexamethasone implants, but demonstrated either a short duration of response or insufficient therapeutic effect.

In this context, FAc implant was considered to be a viable option, given its potential to provide sustained effects for up to 3 years [11, 12]. Various studies have assessed the effectiveness and safety of the FAc implant in patients with PCME [13–24].

Compared to currently available evidence, our case series corroborates positive functional and anatomical outcomes with the FAc implant in PCME, further demonstrating prolonged periods of disease control with a single FAc implant.

**Fig. 5:**
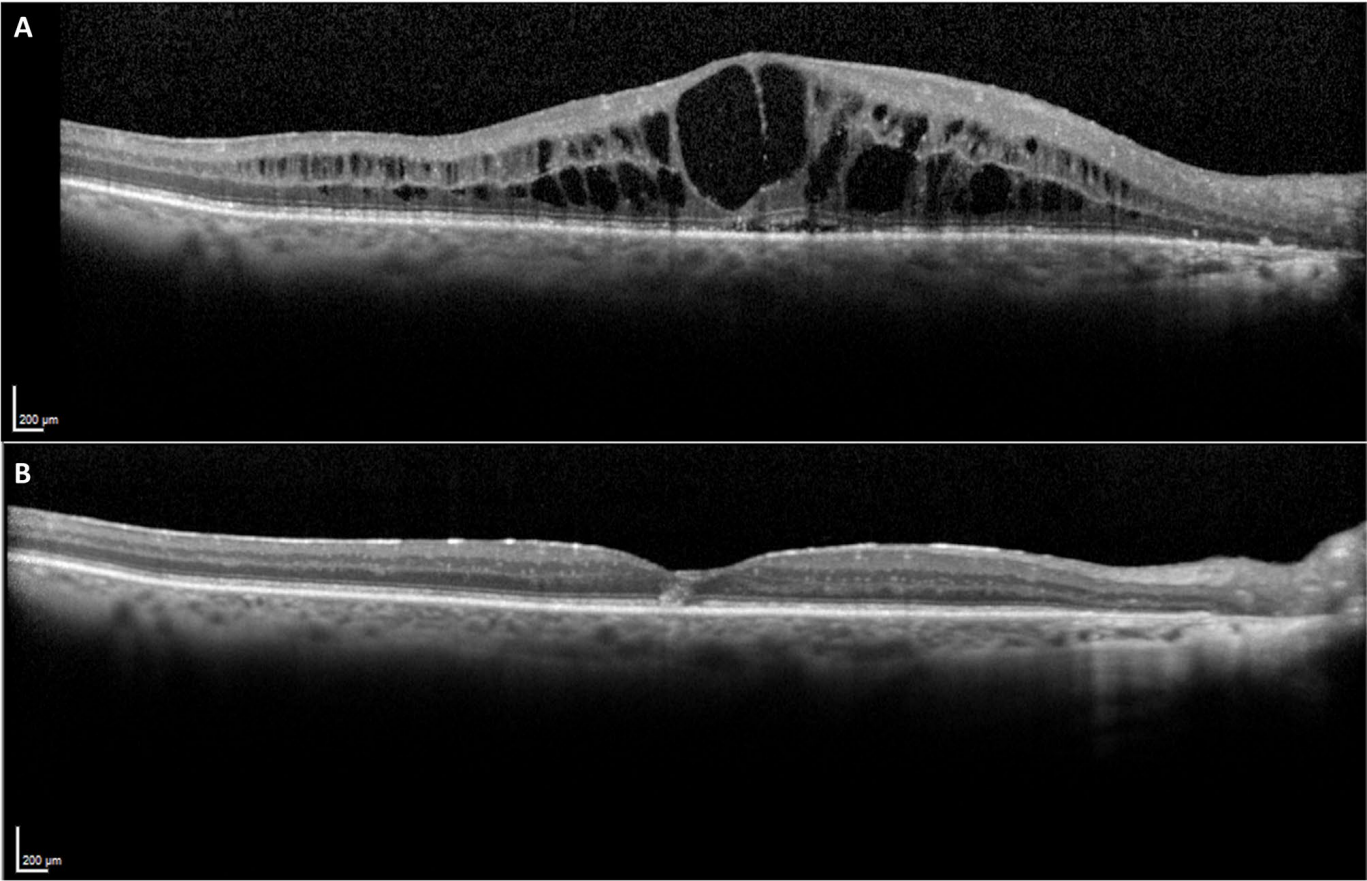
Macular optical coherence tomography at baseline (**A**) and 36 months after FAc implant (**B**) (example from 1 eye included in the study)

The efficacy of topical treatments in eyes who had undergone a vitrectomy is typically constrained [2, 3, 7]. Similarly, anti-VEGF injections often demonstrate reduced effectiveness in vitrectomized eyes due to faster clearance and diminished half-life [2, 3, 7, 29]. Although in vitrectomized eyes intravitreal injections of triamcinolone acetonide injection has shown superiority over anti-VEGF [7], its clearance is much faster than in non-vitrectomized eyes [30, 31]. Conversely, the vitreoretinal pharmacokinetic profiles of FAc implant in vitrectomized eyes mirror those in non-vitrectomized eyes [32]. Albright an insignificant sample size, 1 patient included in this study demonstrated remarkable functional and anatomical outcomes with the FAc implant (25 ETDRS letters gain and 344 μm reduction in CRT, at 36 months follow-up, Fig. 5).

In the current study, no adjunctive therapies were administered following the intravitreal injection of FAc implant. Previous investigations have demonstrated a marked decrease in therapeutic burden post-FAc implantation. For instance, a significant reduction from 2.45 ± 1.35 to 0.57 ± 0.60 DEX implants per year was observed after FAc administration [29]. Consistent with these findings, Lima-Fontes et al. [16] reported sustained control of PCME with a single FAc implant, with recurrence noted in only two eyes over a two-year follow-up period.

The primary complications following intravitreal corticosteroid treatment typically include cataract formation and elevation in IOP. In our series, all treated eyes had undergone cataract surgery prior to receiving the FAc implant, thus precluding the assessment of cataract formation risk. Concerning IOP, one eye had been diagnosed with glaucoma, and seven eyes were receiving ocular hypotensive treatment at baseline due to a hypertensive response to prior steroid therapy. Following the FAc implant, the IOP remained stable throughout the study follow-up period, with no eye experiencing an IOP elevation greater than ≥ 10 mmHg or ≥ 20 mmHg, but 3 eyes had an IOP > 21 mmHg throughout the study. It is noteworthy that at the time of FAc implant administration, 87.5% (7 out of 8) of patients were receiving ocular hypotensive therapy, a finding that may be associated with prior exposure to topical or locally administered corticosteroids in these eyes.

It is important to acknowledge the limitations of this study when interpreting the results, including the retrospective design and the relatively low number of cases included.

## Conclusions

The results of this study clearly indicated that the FAc implant not only successfully managed PCME over the long term, thus averting potential disease recurrences, but also provided long-term visual improvements compared to previous treatments, including the dexamethasone intravitreal implant.

These findings demonstrate that FAc implant emerges as a viable therapeutic alternative for cases of PCME refractory to other therapies. It reduces the need for repeated treatments whilst extending recurrence-free periods following a single injection.

## Data Availability

The datasets used and/or analyzed during the current study are available from the corresponding author [MFM] on reasonable request.
